# Leiomyosarcoma of the rectum mimicking primary ovarian carcinoma: a case report

**DOI:** 10.1186/1757-2215-6-27

**Published:** 2013-04-15

**Authors:** Yung-Taek Ouh, Jin Hwa Hong, Kyung-Jin Min, Kyeong-A So, Jae Kwan Lee

**Affiliations:** 1 Department of Obstetrics and Gynecology, Guro Hospital, College of Medicine, Korea University, Seoul 152-703, Republic of Korea

**Keywords:** Leiomyosarcoma, Rectum, Ovarian carcinoma

## Abstract

Leiomyosarcoma of the rectum is a very rare mesenchymal tumor. Because of its rarity, its diagnosis, treatment, and pathology often present challenges to the clinician. The characteristics of this tumor, such as its anatomical location, heterogeneous solid features on imaging, and nonspecific lower gastrointestinal tract symptoms, can be confused with those of primary ovarian carcinoma. Here, we report the case of a 52-year-old-woman presenting with a low abdominal mass that was later pathologically confirmed to be a rectal leiomyosarcoma. The findings of preoperative ultrasonography, pelvic magnetic resonance imaging, and abdominopelvic computed tomography were suggestive of a malignant pelvic mass, most likely a primary ovarian carcinoma. The patient underwent explorative laparotomy, and intraoperative frozen examination revealed a sarcoma originating from the gastrointestinal tract. Low anterior resection and supracervical hysterectomy with bilateral salpingo-oophorectomy were performed. The patient’s postoperative course was uneventful, and adjuvant chemotherapy is currently being administered.

## Background

Rectal leiomyosarcoma is an uncommon malignancy accounting for less than 0.1% of all malignancies of the colon and rectum [1]. These tumors typically occur in the fifth and sixth decades of life and show a male predominance [2]. The presenting symptoms are rectal pain, constipation, rectal fullness, and diarrhea, although some patients may be asymptomatic [3]. According to the literature, most rectal leiomyosarcomas are identified as protruding masses during colonoscopic examination and are confirmed histologically [4-6]. However, if they are not detected by endoscopic examination, an accurate diagnosis may be difficult because their clinical presentation can mimic that of primary ovarian carcinoma. Here, we report the case of a woman who had a large pelvic mass that was suggestive of a primary ovarian malignancy but was finally proven to be a leiomyosarcoma of the rectum.

### Case description

A 52-year-old woman (gravida 6, para 1) presented to the gynecology clinic at Guro Hospital, College of Medicine, Korea University, with a complaint of low abdominal pain. In addition to low abdominal pain, the patient had experienced low back pain, urinary frequency, and tenesmus for approximately 1 year. She had undergone myomectomy 2 years previously in China.

**Figure 1:**
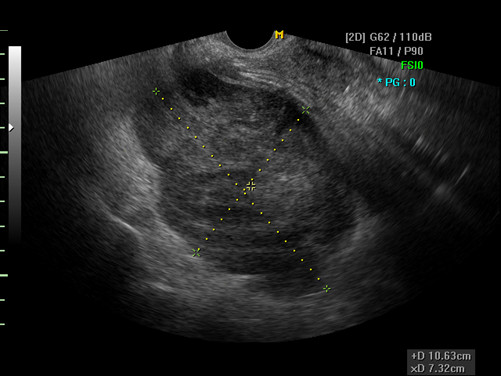
Transvaginal ultrasound showing mixed echogenic, solid mass, measuring 10.6 × 7.3 cm.

**Figure 2:**
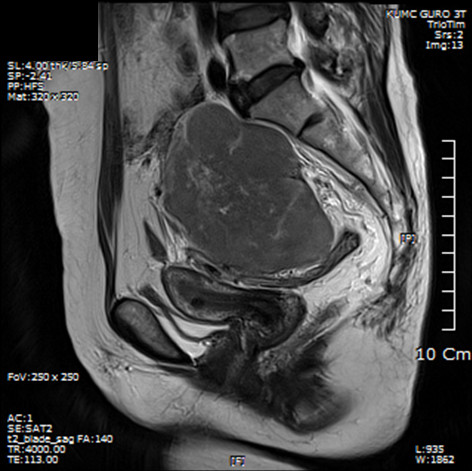
**Pelvic magnetic resonance imaging scan of T2-weighted image.** The mass shows iso- to slightly low signal intensity with heterogeneous enhancement. The uterus and sigmoid colon were displaced anteroinferiorly.

**Figure 3:**
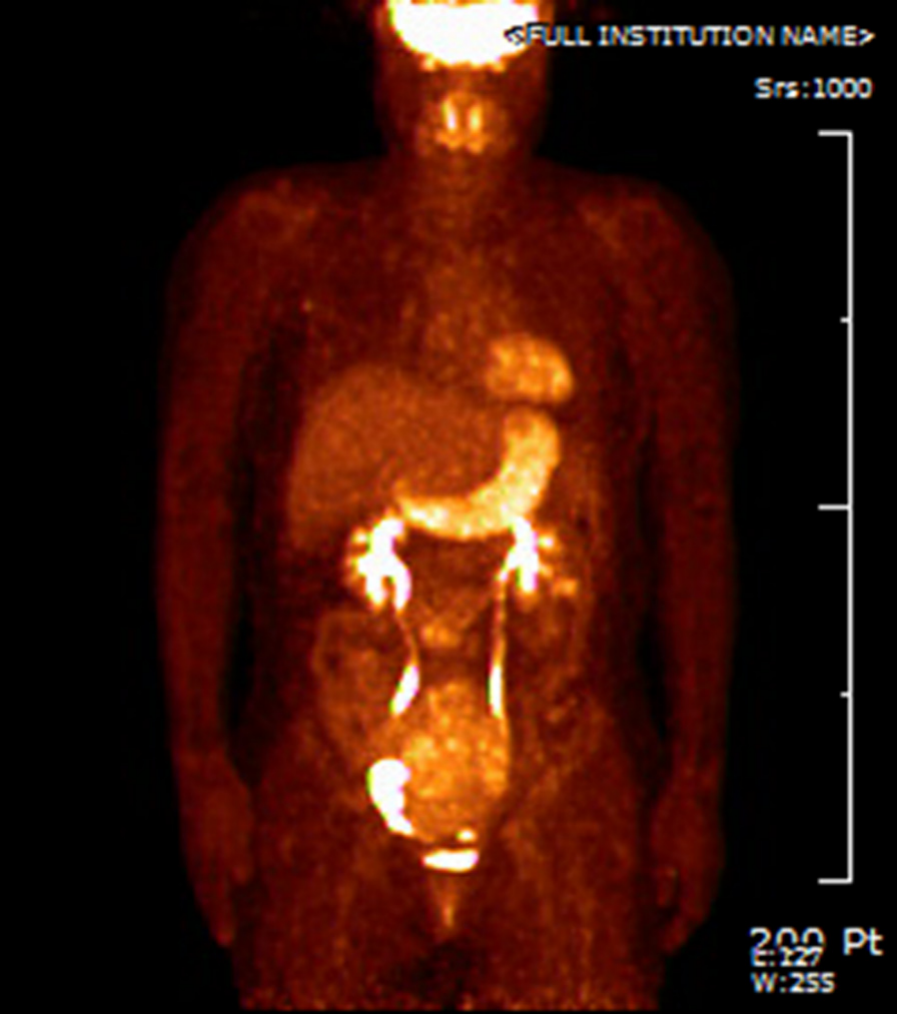
^**18**^**F-fluorodeoxyglucose positron emission tomography/computed tomography scan showing a huge pelvic mass with heterogeneous metabolism.**

The patient’s menses had ceased at the age of 50. She had no complaints of vaginal bleeding or low abdominal distension. She has never undergone a cervical screening test. On pelvic examination, the cervix was found to be small, and no specific abnormalities were observed. On physical examination, a hard mass with an irregular surface was palpable. A Pap smear and a HC2 test were performed. The Pap smear result was a low-grade squamous intraepithelial lesion, and The HC2 test result was negative for high-risk human papillomavirus. Transvaginal ultrasonography examination revealed an approximately 11-cm mixed echogenic solid mass of the left adnexa, which strongly suggested the presence of a malignant tumor (Figure 1). However, serum CA 125 (6.9 U/mL), CA 19–9 (10.8 U/mL), CEA (<0.5 ng/mL), AFP (2.5 ng/mL), and beta-hCG (2.3 mIU/mL) levels were not elevated. The findings of chest radiography, mammography, and breast sonography were normal. An endoscopic examination of the stomach and colon showed no evidence of metastasis. However, colonoscopic evaluation was unsuccessful because of stenosis of the lumen, and the rectal mucosa protruded into the lumen, probably because of extrinsic compression by the pelvic mass. Pelvic MRI and CT of the abdomen and pelvis revealed an approximately 12.4-cm heterogeneous solid mass occupying the entire pelvis and a portion of the lower abdominal cavity. This mass also showed internal degeneration and hemorrhagic change, findings strongly suggestive of a primary ovarian malignancy (Figure 2). The mass was abutted to the posterior sigmoid colon, whereas the presence of direct invasion was indeterminate. The uterus displayed an approximately 1-cm well-defined mass that was suggestive of leiomyoma, but both ovaries were not delineated. On ^18^F-FDG PET/CT, a large pelvic mass with heterogeneous metabolism was identified, suggesting the presence of a malignant mass (Figure 3). No specific finding suggestive of metastasis was observed.

Explorative laparotomy with a supraumbilical incision was performed. A large mass with a longest diameter of approximately 20 cm was identified. This mass was located in the cul-de-sac and was densely adhered to the rectum, retroperitoneal surface, and posterior uterine body. The uterus and both ovaries and tubes were normal on gross appearance. A portion of the mass was subjected to frozen examination, and the result was a mesenchymal tumor originating from the gastrointestinal tract, such as a gastrointestinal stromal tumor. We then performed supracervical hysterectomy and bilateral salpingo-oophorectomy. After the completion of gynecologic surgery, a surgical team from the department of colorectal surgery subsequently performed anterior resection of the colon and appendectomy. The operation was uneventful, and the patient was transferred to the department of colorectal surgery after the operation.

**Figure 4:**
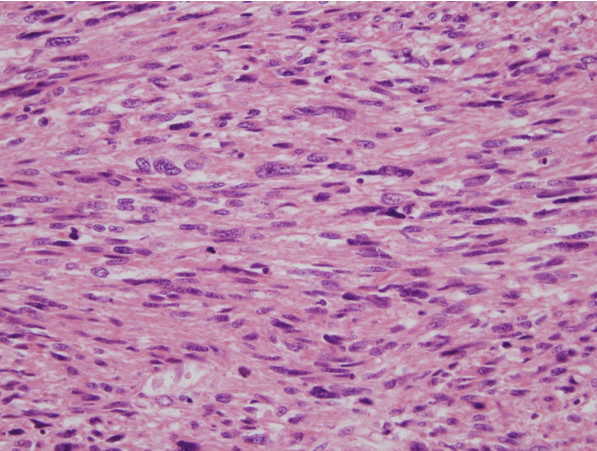
**Microscopic appearance of the resected tumor.** Tumor cells had characteristically elongated, pleomorphic, and blunt-ended nuclei and eosinophilic to pale cytoplasm.

Histopathological analysis of the full specimen revealed a grade 3 leiomyosarcoma, 22 × 17 × 6 cm^3^ in size, with free mucosal resection margins. The mitotic count was ≥20 per 10 high-power fields, and an area of necrosis was identified. The tumor cells had characteristically elongated, pleomorphic, and blunt-ended nuclei and eosinophilic to pale cytoplasm (Figure 4). On immunohistochemical staining, the tumor cells were positive for smooth muscle actin, desmin, and CD99 but negative for S-100 protein and CD34, consistent with a diagnosis of rectal leiomyosarcoma. The resected appendix also showed the presence of a metastatic leiomyosarcoma. The uterus and both adnexa were free of metastasis. Postoperatively, the patient underwent adjuvant chemotherapy with cyclophosphamide, vincristine, and doxorubicin.

## Discussion

Leiomyosarcomas account for 10–20% of all soft tissue sarcomas. They arise most frequently from the uterus, gastrointestinal tract, and retroperitoneal region [7]. Those originating from the rectum are very uncommon, and account for less than 0.1% of all colorectal malignancies [1].

Radiological differentiation between leiomyosarcomas and leiomyomas is difficult, and the final diagnosis needs to be confirmed by postoperative pathological examination. Histological analysis of superficial biopsy samples might not reflect the entire tumor mass and leiomyosarcoma can be misdiagnosed as benign leiomyomas. In addition, malignant potential can be missed on CT of MRI. Given that leiomyosarcoma has a very poor prognosis, misdiagnosis may give a detrimental effect on patient outcome. In this case, all preoperative evaluations, excluding those of serum tumor markers, identified primary ovarian cancer as the most probable diagnosis. To this point, no specific tumor markers have exhibited clinical utility for the diagnosis of leiomyosarcomas. In one series of 10 cases of colorectal leiomyosarcomas, 9 of 10 patients had lobulated tumor margins on CT or MRI [8]. Except in the case of 1 patient, the tumors appeared to be heterogeneous with varying degrees of internal necrosis, findings consistent with those of our case. In 1 patient for whom MRI data were available, the tumor appeared to have intermediate signal intensity on T2-weighted images with heterogeneous contrast enhancement. In another case of rectal leiomyosarcoma, MRI demonstrated a mass of uniform, intermediate signal intensity on T1-weighted images, and heterogeneous high signal intensity on T2-weighted images. Irregular enhancement occurred after intravenous gadolinium [9]. Unfortunately, the aforementioned findings are also commonly encountered in patients with ovarian carcinomas. Therefore, preoperative radiological differentiation between ovarian carcinoma and rectal leiomyosarcoma is difficult.

A distinguishing feature of our case is that the rectal tumor mass was not identified on total colonoscopy. Generally, colonoscopic examination including sigmoidoscopy shows a polypoid, submucosal mass occupying the lumen, leading to the suspicion of a tumor originating in the rectum. In such cases, rectal bleeding or obstruction can occur, prompting an evaluation of the possibility of colorectal problems. However, some rectal leiomyosarcomas growing away from the lumen, so-called exocolic growth, may not be detected on colonoscopic examination. The growth pattern of the tumor and the lack of specific symptoms might be responsible for the failure of preoperative diagnosis.

Pathologically, leiomyosarcomas can be distinguished from leiomyomas on the basis of the following features: larger tumor cells, fewer stromal fibers, increased mitotic activity, and nuclear pleomorphism [10]. Among these findings, the presence of mitoses is the hallmark of malignancy (5 or more mitoses per 10 high-power fields) [11].

The spread of rectal leiomyosarcoma is mainly local or hematogenous, although lymphatic metastasis has been reported in some poorly differentiated tumors [8]. In our case, no metastasis was found in resected perirectal lymph nodes. The optimal treatment modality in patients with rectal leiomyosarcomas is controversial. Wide local excision and radical surgery, such as anterior resection or abdominoperineal resection, are commonly used. As shown in many studies, radical surgery is associated with a lower recurrence rate than wide local excision [2,12]. However, differences in survival rates were not statistically significant, regardless of the treatment modality [2,12]. Pelvic radiation therapy is generally considered unsuccessful. Minsky et al. reported moderate success using radiation therapy following surgery [13]. In contrast, Consentino et al. demonstrated that neither adjuvant radiation therapy nor chemotherapy is effective [14]. Chemotherapy has been generally unsuccessful in treating this tumor. The 2 most commonly used agents, doxorubicin and dacarbazine, are associated with low response rates ranging from 15% to 30% [15]. Unfortunately, there are few data regarding the efficacy of adjuvant treatments in the literature to draw definitive conclusions. The overall prognosis is poor, with reported survival of 20–40% at 5 years and significant additional mortality reported during later years [16]. Tumor size and the degree of differentiation are known to be the most significant prognostic factors.

## Conclusion

In this study, we presented a case of rectal leiomyosarcoma mimicking primary ovarian carcinoma. We can draw a lesson from this case report. When a woman presents with a pelvic mass that is suggestive of ovarian carcinoma on radiological evaluation but is suggestive of ovarian cancer on the basis of normal serum tumor marker levels, the possibility of rectal leiomyosarcoma should be suspected even in the absence of rectal bleeding or pain. We also caution that appropriate surgical treatment should not be delayed solely on the basis of normal tumor marker levels. Because of its poor prognosis, early diagnosis and prompt surgical removal are important for patients with rectal leiomyosarcoma.

### Consent

Written informed consent was obtained from the patient for publication of this case report and any accompanying images. A copy of the written consent is available for review by the Editor-In-Chief of this journal.

## Abbreviations

Pap: Papanicolaou; HC2: Hybrid Capture 2; CA 125: Carbohydrate antigen 125; CEA: Carcinoembryonic antigen; AFP: Alpha fetoprotein; beta-hCG: beta-human chorionic gonadotropin; MRI: Magnetic resonance imaging; CT: Computed tomography; FDG: fluorodeoxyglucose; PET: Positron emission tomography; CD: Cluster of differentiation.

## Competing interests

The authors declare that they have no competing interests.

## Authors’ contributions

YTO drafted the manuscript. JHH supervised the study. KJM and KAS were involved in design and acquisition of data. JKL revised the manuscript and gave final approval of the version to be published. All authors read and approved the final manuscript.
